# Supplementary figures and images for: In vivo neural activity of electrosensory pyramidal cells: Biophysical characterization and phenomenological modeling
Source: PLoS Comput Biol. 2025 Nov 17;21(11):e1013711. doi: 10.1371/journal.pcbi.1013711 (PMC12633970; doi:10.1371/journal.pcbi.1013711)

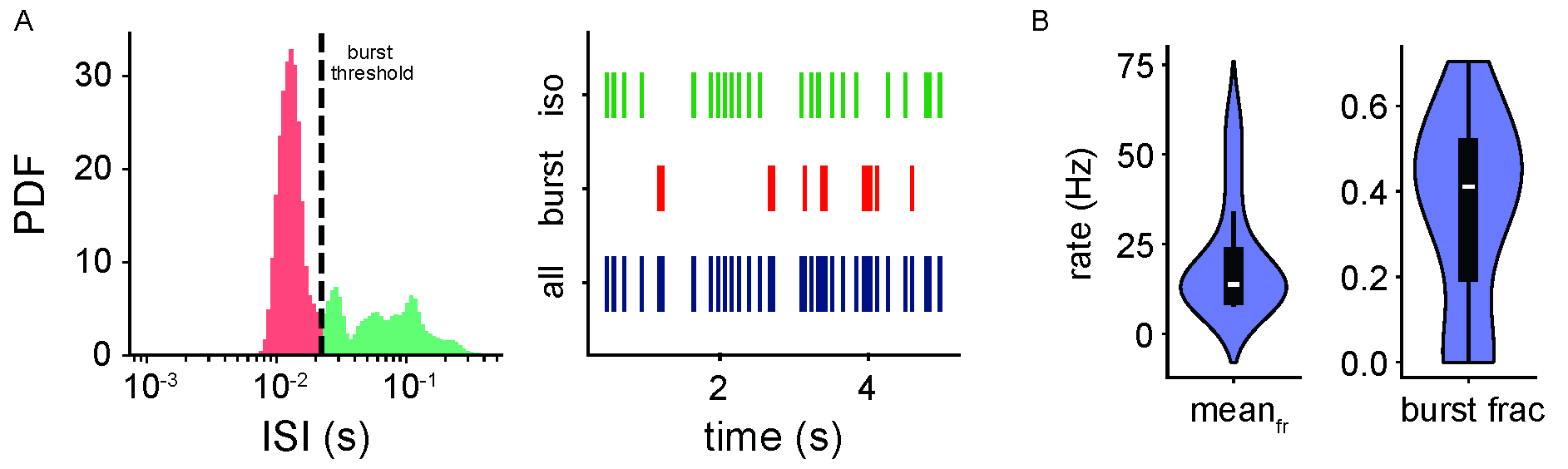

Supplement: S1 Fig — (A) Illustration of burst definition and spike train separation for a representative neuron. Left: The burst threshold (dashed line) is set at the local minimum, separating short ISI mode (red) from longer ISIs associated with isolated spikes (green). Right: Spike raster plot of the same cell showing all recorded spikes (dark blue), and the resulting separation into burst spikes (red) and isolated spikes (green) based on the ISI threshold. (B) Distribution of mean firing rates (left, computed from all spikes over the entire recording duration) and burst fractions (right) for all recorded pyramidal cells (N = 8 fish, n = 32 cells). Each intracellular recording lasted 5 sec. (TIFF) [file pcbi.1013711.s001.tif]

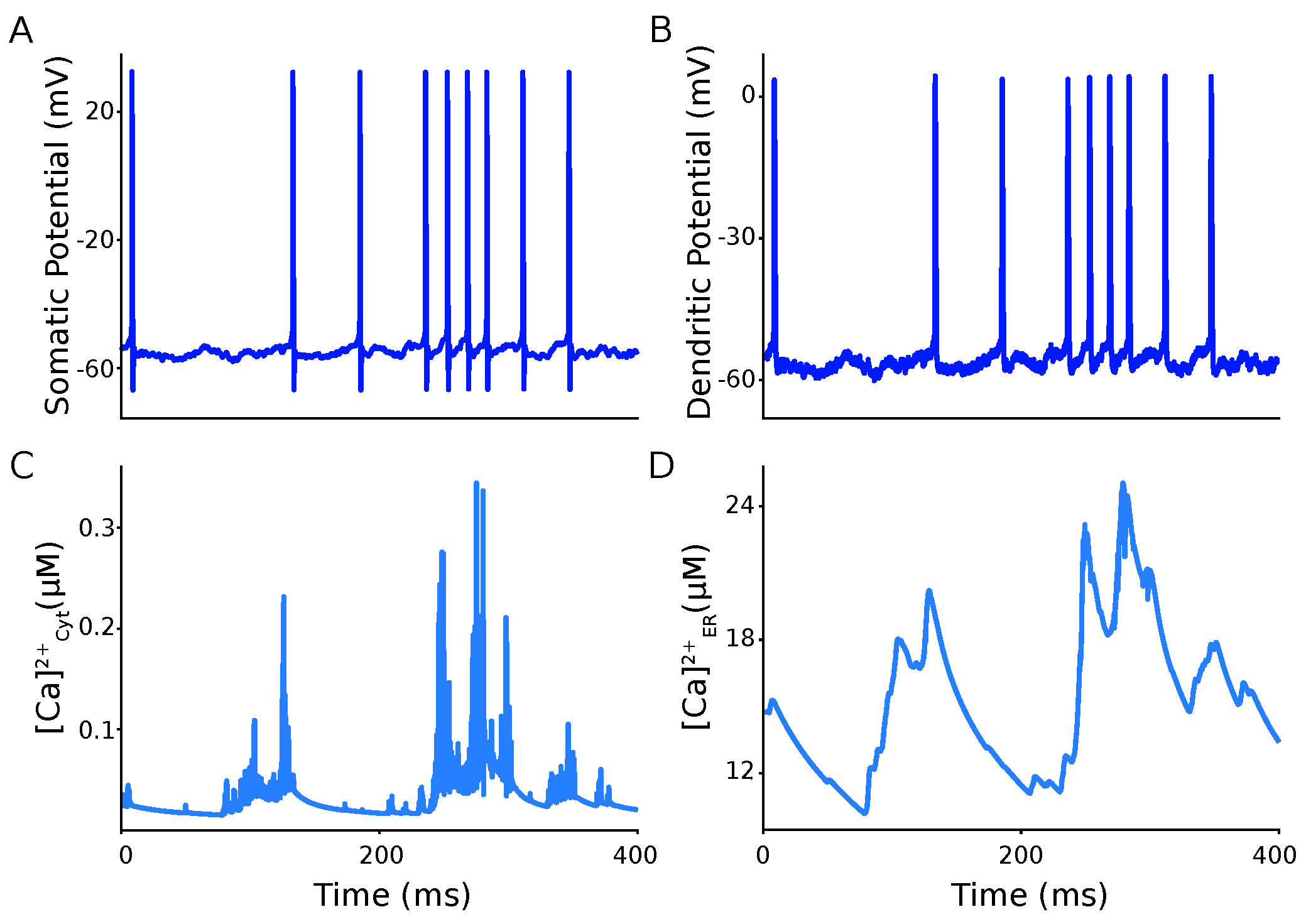

Supplement: S2 Fig — (A, B) Somatic (A) and dendritic (B) membrane voltages. The somatic action potential backpropagates to the dendritic compartment, generating dendritic action potentials upon activation of Na + channels in the dendrite, which in turn propagates to the soma through electrodiffusion coupling. Spike train traces show a gradual decrease in consecutive ISIs during the burst period but no evidence of the depolarizing afterpotential (DAP) growth or failure in the dendritic action potentials upon the termination of the burst, similar to in vivo recordings. (C, D) Time series of the Ca2 + concentration within the cytosol (C) and ER (D) from the dendritic compartment. Ca2 + release into the dendritic membrane through NMDAR or via the ER activates SK channels, which in turn promotes afterhyperpolarization in the membrane potential. This mechanism is the primary driver of DAP termination, preventing prolonged dendritic spike backpropagation and accounting for the difference in the burst mechanism between in vitro and in vivo recordings. (TIFF) [file pcbi.1013711.s002.tif]

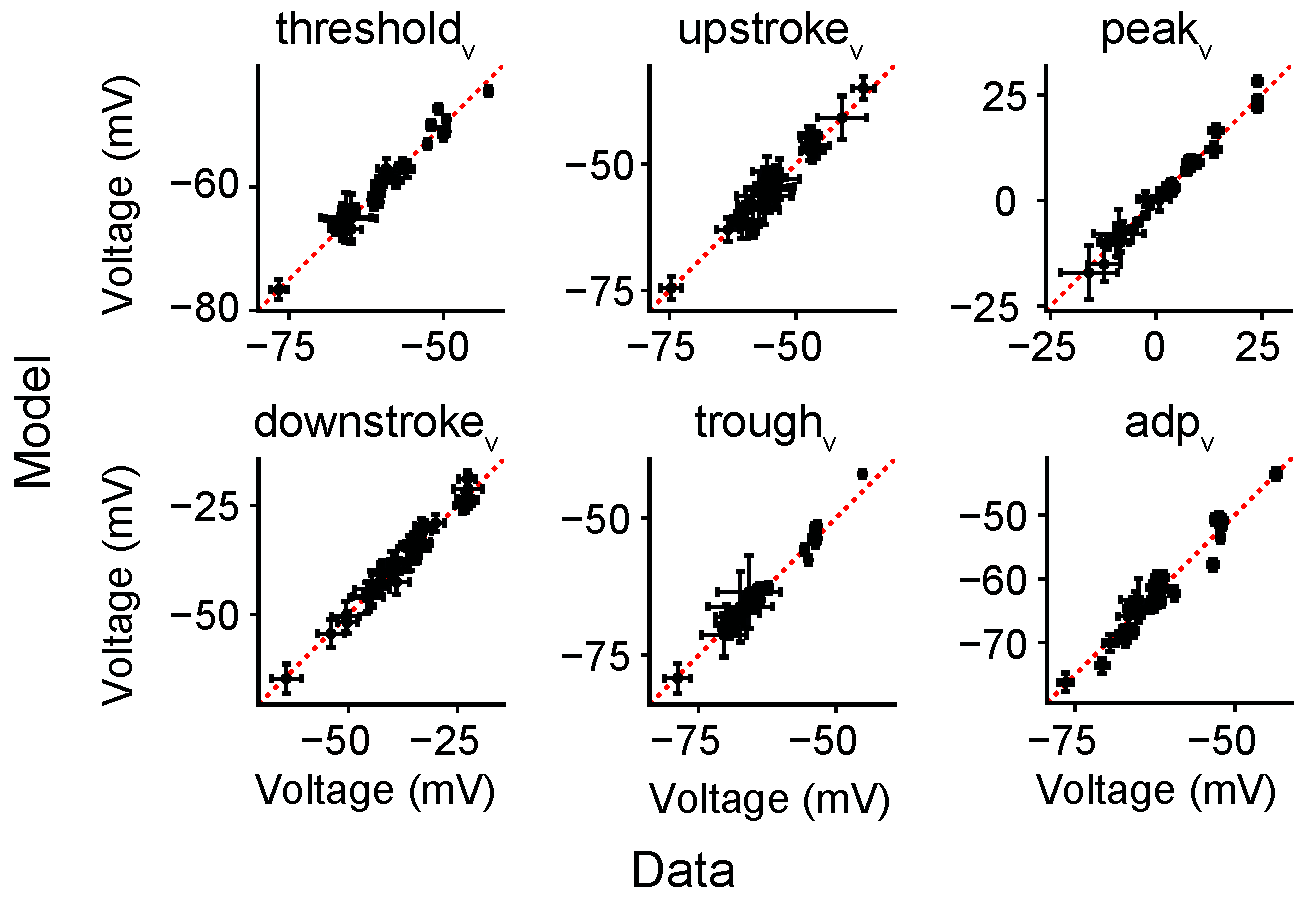

Supplement: S3 Fig — Scatter plots comparing the mean value of each action potential feature between experimental data (data) and corresponding fitted model simulations (model) for all recorded ELL pyramidal cells (n = 32). Features shown from left to right : spike threshold (thresholdv), midpoint of the upstroke phase (upstrokev), peak amplitudes (peakv), midpoint of the downstroke phase (downstrokev), trough amplitudes (troughv) and amplitude of afterdepolarization potential (adpv). Points cluster closely around the identity line, indicating strong agreement (linear fit: rthreshold=0.98, pthreshold=3.7×10^−23^; rupstroke=0.97, pupstroke=4.6×10^−21^; rpeak=0.99, ppeak=1.0×10^−27^; rdownstroke=0.98, pdownstroke=1.0×10^−26^; rtrough=0.98, ptrough=2.7×10^−24^; radp=0.97, padp=1.7×10^−20^). Error bars indicate standard errors (SEM). (TIFF) [file pcbi.1013711.s003.tif]

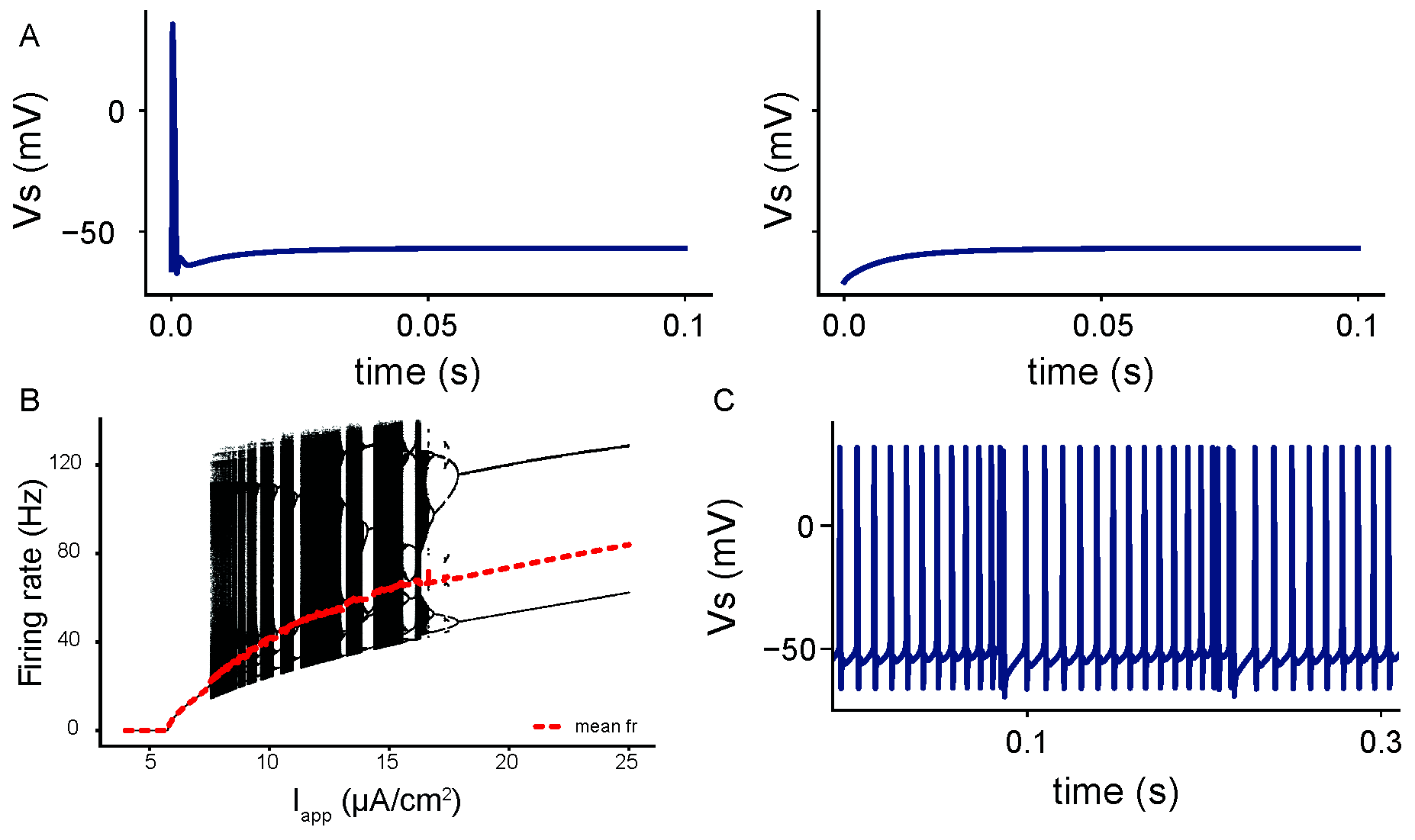

Supplement: S4 Fig — (A) Membrane potential simulations of the biophysical model (0.1 s duration) with identical parameter values but different initial conditions, using a subthreshold value of the depolarizing current (Iapp) below the SN1 bifurcation point (see Fig 4A). Depending on the initial condition of the system, the model either returns directly to the resting state (right panel) or generates a single transient spike before returning to rest (left panel). (B) The firing rate of the biophysical two-compartment model detected within a time range of 20 seconds with respect to Iapp, showcasing the chaotic dynamics exhibited by this model. The red curve represents the average firing rate. (C) Simulation of the biophysical model after blocking SK and NMDA currents gSK = 0, gNMDA = 0). In the absence of these currents, the model exhibits prolonged bursts with prominent depolarizing afterpotentials (DAPs), reverting to dynamics characteristic of the original in vitro ghostbursting model. (TIFF) [file pcbi.1013711.s004.tif]

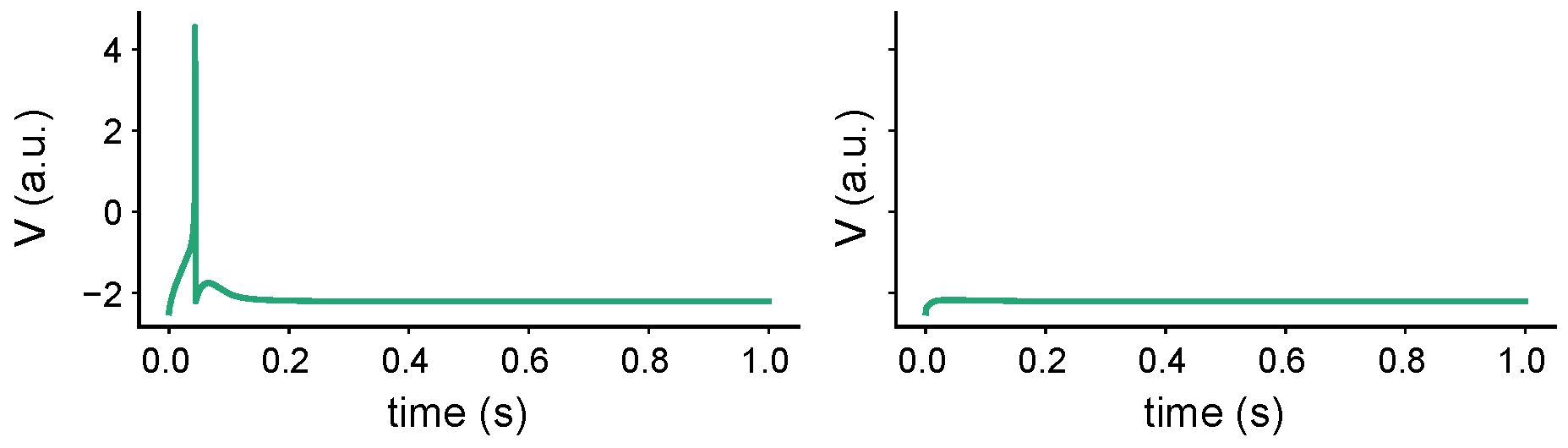

Supplement: S5 Fig — Membrane potential simulations (1s duration) for the same parameter values using two different initial conditions with the applied current Iapp below the spiking threshold. Depending on the specific initial condition, the model either generates a single transient spike before returning to rest (left) or returns directly to the resting state without spiking (right). (TIFF) [file pcbi.1013711.s005.tif]

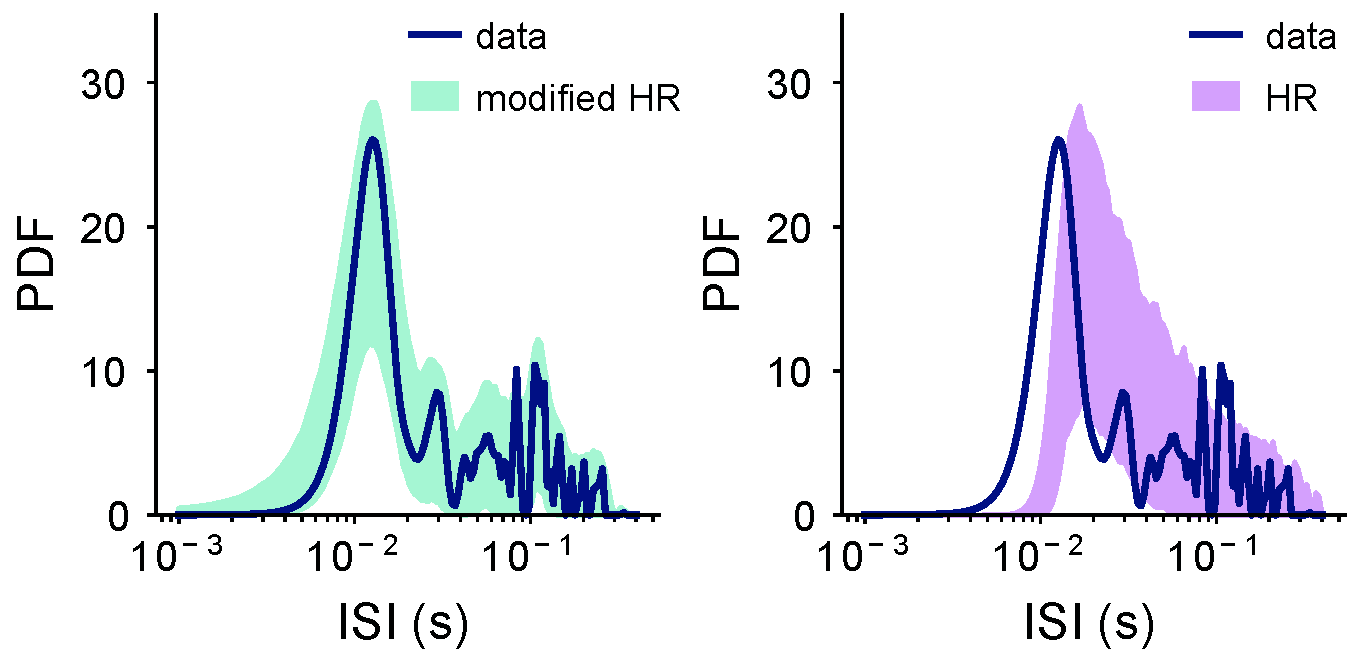

Supplement: S6 Fig — Comparison of the interspike interval (ISI) distributions between an example recorded ELL pyramidal cell (blue) and ISIs from model simulations of modified HR model (teal, left) and classic HR model (purple, right) both having same parameter values and stochastic synaptic input (Kolmogorov-Smirnov test vs data: DmodifiedHR=0.146, pmodified HR=0.778; DHR=0.383, pHR=0.0065). (TIFF) [file pcbi.1013711.s006.tif]
