# Supplementary material for: In vivo neural activity of electrosensory pyramidal cells: Biophysical characterization and phenomenological modeling
Source: PLoS Comput Biol. 2025 Nov 17;21(11):e1013711. doi: 10.1371/journal.pcbi.1013711 (PMC12633970; doi:10.1371/journal.pcbi.1013711)
Supplement: Attachment — Submitted filename: Rebuttal_letter_PCOMPBIOL-D-25-01154.pdf [file pcbi.1013711.s010.pdf]

Reply to Reviewers for PCOMPBIOL-D-25-01154

In vivo neural activity of electrosensory pyramidal cells

Akhshi et al.

October 3, 2025

Reviewer #1

The current manuscript investigates how burst firing in electrosensory pyramidal cells of the electrosensory lateral line lobe (ELL) in the weakly electric fish *Apteronotus leptorhynchus* is generated and modulated. The authors combine intracellular recordings with computational modeling to study the activity of these neurons. They first constructed a detailed biophysical, two-compartment model based on in vivo data, incorporating intrinsic ionic conductances, SK channel activation, calcium dynamics, and stochastic synaptic inputs. Using bifurcation analysis, they show that these parameters govern transitions between quiescent, tonic, and bursting firing modes. The authors further developed a phenomenological model based on the Hindmarsh-Rose (HR) formalism, demonstrating that this modified HR model can reproduce ELL neuronal activity. Both models are compared and validated against the in vivo recordings. Finally, a parameter sensitivity analysis highlights the importance of slow adaptation and stochastic input in shaping ELL neurons' spiking patterns and provides a compelling hypothesis to explain the differences between in vivo and in vitro recordings of these neurons. The model is both detailed and valid, and compared rigorously with the in-vivo data that have been recorded for the project. However, there are some limitations on the experimental side that might greatly weaken some of the conclusions made in the manuscript.

Reply: We sincerely thank the reviewer for their positive assessment of our manuscript and have made every effort to address all the concerns raised.

—

Major Points

Comment: The neurons measured in the in-vivo model animals are described as being recorded in absence of stimulation. However, the authors specify that "ELL pyramidal cells receive direct ascending inputs from electroreceptor afferents": were there any controls in the experimental setup that made sure electroreceptors were not activated, or that there was no electric field that could have been detected? If not, could the authors please explain why it is not necessary to make such control? While my expertise is with rodens models, I understand that weakly electric fish self-generate electric fields and use them to sense nearby objects: their electroreceptors being activated

by these self-generated currents. Were these currents being measured, or can we be sure they could not have been present due to the condition the animals were in? If potential external sensory stimuli can not be ruled out, this could mean that the importance of intrinsic noise could have been (potentially greatly) overstated, therefore impacting the main conclusions of the study.

Reply: We now clarify in the text that the "absence of stimulation" condition refers to the baseline electrosensory state in which the animal's own electric organ discharge (EOD) is present but unmodulated. It is important to note that the EOD serves only as a carrier signal and does not by itself constitute sensory input; only perturbations of the EOD (e.g., caused by objects, conspecifics, or externally applied fields) generate amplitude modulations that are detected by electroreceptors. In our experiments, immobilized animals were housed individually in isolated tanks during recordings, which ensured the absence of such perturbations and minimized environmental noise. This is the standard condition for *in vivo* intracellular recordings in weakly electric fish, and has been used in multiple prior studies. Thus, the baseline activity we analyzed reflects intrinsic and synaptic mechanisms rather than external sensory drive. We have added this clarification to the Results (Lines 107–113) and Methods Section (Lines 745–751).

—

Comment: The experimental part of the study suffers from a low number of animals the recordings were taken from. While a preferable solution would be to improve the number of recorded fish, I would at least recommend the authors to clearly show the variability of the data across the 3 fish they recorded from. One way to do this would be to plot AP-related electrophysiological characteristics for the 3 fish. Showing that these characteristics are consistent across animals would greatly improve the reliability of the in-vivo results. Further, and closely related to the previous point, a common source of biased results when averaging over all the cells from multiple animals together, happens when the number of recorded cells greatly fluctuates between animals: can the authors please specify how many cells they recorded from each animal?

Reply: We have now increased our dataset from $N = 3$ fish ($n = 20$ cells) to $N = 8$ fish ($n = 32$ cells) and report the number of cells recorded from each fish in the Methods Section. To address the concern regarding variability across animals, we performed a per-fish analysis of AP-related features (threshold, upstroke, peak, downstroke, trough, ADP). We used intraclass correlation coefficients (ICC) to assess the source of variability in AP-related features inter vs. intra fish. We found that the majority of variance arises within fish rather than between fish (e.g., $\mathrm{ICC}_{\mathrm{threshold}} = 0.33$; $\mathrm{ICC}_{\mathrm{upstroke}} = 0.04$; $\mathrm{ICC}_{\mathrm{peak}} = 0.31$; $\mathrm{ICC}_{\mathrm{downstroke}} = 0.39$; $\mathrm{ICC}_{\mathrm{trough}} = 0.26$; $\mathrm{ICC}_{\mathrm{ADP}} = 0.49$). These results indicate that cell-to-cell variability dominates over animal-to-animal variability. We have revised the Methods Section (Lines 759–773) to clarify these points. Finally, it is important to note that the sensitivity and bifurcation analyses conducted in this study further provide insights into how these variabilities are generated and how they may affect dynamics.

—

Minor Points

Comment: Since one of the objectives of the model is to capture bursting behavior, I believe it would be beneficial to show a comparison between a typical burst as recorded in-vivo, and a burst

generated by the model. Though this is partly done in Figure S3, I believe this is important to show in one of the main figures. The most straightforward way to do this would probably be to make a small addition to Figure 3, showing via a "zoom in", the typical shape of a recorded vs generated burst.

Reply: As suggested by the reviewer, we have added insets to Fig. 3A showing zoomed-in experimental and simulated traces, to better illustrate how bursting compares between them. We accordingly modified the text (Lines 219–222) in the Results Section.

—

Comment: In Figures 3 and S3, the authors plot the electrophysiological characteristics of recorded vs generated action potentials, and associated action potential trains. While it is clear that the model correctly represents the experimentally recorded data, it would be beneficial to also compare the variability between the two samples. A way to do this might be to add error bars (on both axis) to these plots, or make separate plots in addition to Figure S3.

Reply: As requested, we have added the standard errors (SEM) to Figure S3.

—

Comment: In Figure S1B, the authors plot the excitatory neurons mean firing rate, however, from the context it is difficult to understand if the firing rate is relative to only bursts, or to the entire recordings. Further, the length of the recordings is not specified (or I can not find it in the methods or figure legends).

Reply: In Supplementary Fig. S1B, the mean firing rate was calculated from all spikes across the entire recording, not only burst spikes. We have clarified this in the figure legend. We also now specify in the Methods Section (Lines 759–761) and figure legend that each recording was 5 seconds in duration, and that all spike train statistics (firing rate, burst fraction, ISI distributions) were computed over the entire recording period.

—

Comment: Flow and readability: at line 36, the authors write: "The in vivo recordings, on the other hand, show markedly different firing patterns, typically consisting of only 2–3 spikes per burst on average, with no evidence of a somatic DAP or burst AHP". The in vitro vs in vivo comparison is a key point of the paper, and the authors do mention DAP and AHP, however, adding a clearer description of how many APs are typical in an in-vitro burst would help the reader in following the text.

Reply: As suggested by the reviewer, we have now clarified the text by explicitly stating (Lines 53–61) that *in vitro* bursts in ELL pyramidal cells typically consist of 5–8 action potentials on average, accompanied by progressive depolarizing afterpotentials (DAPs) and a prominent burst afterhyperpolarization (AHP). This addition provides a clearer contrast with the shorter 2–3 spike bursts observed *in vivo*.

Reviewer #2

This manuscript combines intracellular recordings and computational modeling to investigate burst firing in ELL cells of weakly electric fish. A biophysically detailed model and a modified HR model reveal that in vivo burst firing arises from interactions among NMDA receptors, SK channels, calcium dynamics, and synaptic noise. The findings explain variability in firing patterns and help reconcile differences between in vivo and in vitro observations.

—

Comment 1: With increasing I_{app}, the biophysically detailed ELL cell model shows a gradual transition from quiescence to tonic firing and then to bursting (Supplementary Fig. 4B), whereas the modified HR model exhibits an abrupt change from quiescence to burst firing (Fig. 5B). I am curious what type of firing pattern do actual ELL cells exhibit with increasing I_{app}?

Reply: We thank the reviewer for this comment. In the original submission, the tonic regime of the modified HR model was not clearly visible. We have increased the resolution of the applied current sampling in Fig. 5C, which now makes this transition clearer. The text has been revised (Lines 394–399) to state explicitly that both the biophysical model (Supplementary Fig. 4B) and the modified HR model (Fig. 5C) exhibit the same sequential transitions from quiescence to tonic firing and then to bursting as I_{app} is increased. With respect to experimental data, our study focused on the spontaneous *in vivo* activity of ELL pyramidal cells and did not involve current step injections. Therefore, we cannot directly report how firing patterns evolve with increasing I_{app} in our recordings. However, previous *in vitro* studies of ELL pyramidal cells (e.g., Rashid et al., 2001; Doiron et al., 2002; Deemyad et al., 2011) have shown a progression from quiescence to tonic spiking and then to bursting as the injected current is increased. We have addressed this in detail in the Discussion (Lines 540–545).

—

Comment 2: Related to the above: as stated in the manuscript, factors such as synaptic inputs and neuromodulators can modulate neuronal responses such as firing patterns. Is it possible that these factors could shift a single cell's firing pattern between two firing patterns represented by biophysically detailed ELL cell model and modified HR model? Or shifting the intrinsic neuronal mechanisms?

Reply: We appreciate the reviewer's comment and agree that synaptic inputs and neuromodulators can directly shift neuronal firing patterns. As noted above (see reply to Comment 1), there is no discrepancy between the firing patterns represented by the two models. We have revised the Discussion (Lines 624–631) to state explicitly that changes in synaptic drive or modulation of SK/NMDA conductances can shift a cell between quiescent, tonic, and bursting regimes, and that both models are capable of capturing these transitions.

—

Comment 3: I understand that various types of models can be fitted to reproduce key features of a particular neuron type, and bifurcation analysis is often used to uncover potential intrinsic mechanisms of this type of neuron. The authors have explained why two models with distinct

firing patterns are used to represent ELL cells in lines 225–238. However, I'm still trying to understand why (and how) two different underlying mechanisms, demonstrated by the respective bifurcation diagrams of two models, contributes to understand *in vivo* behaviors of one type of neuron (i.e., ELL cells). A brief and straightforward explanation would help readers like me better grasp the motivation behind this approach. Additionally, a short summary comparing the common observations, differing conclusions, and limitations of the two models would be very helpful.

Reply: We thank the reviewer for this comment. Our motivation for using both a biophysically detailed model and a phenomenological modified HR model was twofold. The detailed model provides mechanistic insight into how specific conductances (e.g., SK, NMDA) and calcium dynamics shape ELL pyramidal cell activity, whereas the modified HR model offers a simplified, computationally efficient framework that reproduces the same repertoire of firing patterns. Although the bifurcation structures have some differences, both models capture the ghostbursting mechanism, the sequential transitions between quiescence, tonic firing and bursting, as well as the irregular dynamics near bifurcation cascades. We have revised the Discussion (Lines 527 – 539) to provide a short summary comparing the two approaches: both reproduce the essential *in vivo* features (e.g., short bursts, variability, chaotic dynamics), but the biophysical model highlights the role of specific ionic currents and calcium stores, while the modified HR model emphasizes general dynamical principles and could be beneficial for future studies of large networks. We also state explicitly the limitations of each: the biophysical model is computationally expensive, while the modified HR model abstracts away mechanistic detail. This dual approach strengthens our conclusions by showing that the observed *in vivo* burst dynamics are robust to different levels of modeling abstraction.

—

Comment 4: Lines 267–269 state that the modified HR model shows sensitivity to initial conditions. Additionally, the bifurcation analysis for both models has metastability regions. If I understand correctly, this suggests the coexistence of multiple stable states depending on initial conditions. Does this sensitivity/dependence of initial conditions have any biophysical relevance to actual ELL cells or other cells in other systems in general?

Reply: We thank the reviewer for raising this point. The sensitivity to initial conditions and multistability seen in both models are not merely mathematical artifacts but have biological relevance. Indeed, experimental studies in other systems have demonstrated bistability in real neurons, such as dendritic Ca^{2+} dynamics in Purkinje cells (Loewenstein et al., 2005), supporting the idea that multistability can be accessed depending on prior activity and perturbations. *In vivo*, ongoing synaptic input and intrinsic noise act as perturbations that move neurons between coexisting attractors, allowing flexible switching between quiescent, tonic, and bursting regimes. We have revised the Discussion (Lines 655–665) to highlight the potential functional roles for this multistability, as well as its possible contribution to irregular or pathological activity (Lines 698–700), suggesting a general biophysical principle underlying adaptive neuronal computation.

—

Comment 5: Line 321–322: "we fixed all parameters of the deterministic modified HR model based on the SAME EXAMPLE ELL pyramidal cell used earlier and simulated its activity with and without stochastic synaptic noise (Fig. 7B)." (a) Does this mean the authors selected a specific set of parameter values that best fit the recording of this particular example ELL cell? (b) For the biologically detailed ELL model in Figs. 2 and 3, were a fixed set of parameter values used, or

did the parameter values vary across the different recorded ELL cells ($n = 20$)? (c) For modified HR model in Fig. 6, were a fixed set of parameter values used, or did the parameter values vary across different recorded ELL cells? (d) If different sets of parameter values are used for different ELL cells, could the authors clarify what the parameter values are used for bifurcation analysis in Figs. 4 and 5?

Reply: We thank the reviewer for this comment. (a) For the stochastic simulations shown in Fig. 7B, we fixed the deterministic modified HR model parameters to the set obtained from the same example ELL pyramidal cell used earlier (Figs. 6A–C), i.e., the parameters that best reproduced the spike train features of that recording. (b) For the biophysical model (Figs. 2–3), parameter values were optimized individually for each recorded cell ($n = 20$, now $n = 32$ in the revised dataset – see reply to Comment 2, Reviewer 1), so that each cell had a distinct parameter set reproducing its AP waveform and spike train features (see Supplementary Fig. 3). (c) For the modified HR model in Fig. 6, we likewise used multiple parameter sets, each optimized to reproduce spike train statistics of different recorded cells. This allowed us to capture the variability observed across the population. (d) For the bifurcation analyses in Figs. 4 and 5, we used a fixed representative parameter set that was optimized to fit a typical example cell. This ensured consistency of the bifurcation diagrams while keeping the analysis tractable. The full parameter sets used for continuation are now provided in the Supplementary Information and the code repository referenced in the Methods (Lines 963–981).

—

Comment 6: How is the amplitude of afterdepolarization potential (adp_v) in Fig. 2D calculated for the first action potential (or the first several APs) in a burst (such as those in Fig. D3 and D4 for simulation) where the adp_v is not clearly observable in either simulation or recordings?

Reply: The amplitude of the afterdepolarization potential (adp_v) was computed following the Allen Institute's IPFX protocol, which we implemented in our feature extraction pipeline. After each spike's threshold, peak and trough were detected, the algorithm searched the post-spike segment for a local depolarizing maximum (the ADP). The adp_v value reported in Fig. 2D corresponds to the absolute membrane potential at this detected maximum. Importantly, when no ADP maximum could be identified (i.e., for the first one or two spikes in short *in vivo* bursts where no clear ADP hump is visible), the algorithm returned NaN for that spike's adp_v, and such spikes were automatically excluded from ADP statistics. Likewise, spikes clipped at the edges of the analysis window were excluded. This ensures that ADP values are only reported when a measurable depolarization is present and avoids biasing the results with artificially low or absent ADPs. We have now added this explanation to the Methods section (Lines 919–826).

—

Comment 7: Modified HR model on lines 640–643: (a) according to lines 655–656, dW terms in four equations (29)–(33) appear to be different, please correct the mathematical expressions to reflect this. (b) Equation number (32) is missing.

Reply: Fixed.

Reviewer #3

This is a technically sound manuscript that addresses how to reconcile in vivo recordings from ELL pyramidal cells in weakly electric fish with in vitro data, using both biophysically detailed and phenomenological neural models. The electrosensory system is relatively small and well characterised, making it an excellent candidate for mechanistic modelling. The authors combine electrophysiological recordings, computational simulations, and bifurcation analyses to investigate how background synaptic activity, intracellular calcium dynamics, and membrane conductances interact to shape burst firing. While the experimental, modelling, and bifurcation analyses appear to be carefully executed – despite some issues noted below – the manuscript still contains minor omissions, technical inconsistencies, and linguistic errors that require attention. A more thorough revision is needed to improve clarity and presentation. Overall, the simulations offer interesting insights into discrepancies between in vitro and in vivo ELL electrophysiology, particularly regarding bursting regimes. The proposed mechanism, based on the interplay between noise and multistable dynamics, provides an intuitive and potentially valuable framework for understanding this phenomenon. If the text is revised to address the specific points raised below, I believe it would meet the standards for publication in PLoS CB.

Reply: We appreciate the reviewer's careful reading, insightful comments, and positive feedback on the manuscript's suitability for publication after revisions. We have made every effort to address the comments and improve clarity. —

Major

Comment: L13–15: Background synaptic activity does not necessarily lead to a depolarised bias. While it introduces membrane potential fluctuations and can enhance responsiveness, the net effect on membrane potential depends on the balance of excitation and inhibition. Typically, it leads to a high-conductance state with increased variability, not necessarily a shift in mean voltage. See Destexhe et al., 2003; Shu et al., 2003; Haider & McCormick, 2009; Richardson, 2004.

Reply: We thank the reviewer for pointing this out. We now describe background synaptic input as producing a high-conductance state with enhanced fluctuations and responsiveness, without necessarily causing a net depolarization (Lines 26–40), citing Destexhe et al. (2003), Shu et al. (2003), and Haider & McCormick (2009). We further mention that, in the electrosensory system, blockade of background synaptic input decreases firing activity *in vivo* (Bastian and Nguyenkim, 2001; Chacron and Bastian, 2008).

—

Comment: L98–104 and discussion on chaotic bursting. How do your results compare to [Falcke, M., Huerta, R., Rabinovich, M. I., Abarbanel, H. D., Elson, R. C., & Selverston, A. I. (2000). Modeling observed chaotic oscillations in bursting neurons: the role of calcium dynamics and IP 3. Biological cybernetics, 82, 517–527]?

Reply: We appreciate the reviewer drawing our attention to Falcke et al. (2000). We now discuss this study in the Results/Discussion section (Lines 328–333; Lines 571–582), highlighting the common role of calcium dynamics in generating chaotic bursting and contrasting the IP_3-

mediated mechanism they describe with the NMDA/SK-dependent mechanism that emerges in our model.

—

Comment: Please provide more details on the numerical continuation: it is notoriously hard to continue fast-slow dynamics, such as bursting orbits, and yet you seem to track them just fine (dashed green lines on Figs 4,5).

Reply: We used AUTO-07p to perform the bifurcation analysis (Figs. 4 and 5). The revised manuscript describes the numerical constants used in the bifurcation analysis (see Methods, "Bifurcation and Sensitivity Analysis"). Furthermore, we have included the AUTO scripts for performing the bifurcation on GitHub and provided a link in the text.

—

Comment: L125, Fig. 2D: t-tests assess mean differences only, but the boxplots suggest full distributions are of interest. Consider also reporting distributional metrics or using nonparametric tests.

Reply: In addition to the original two-sample t-tests, we now include nonparametric comparisons using Mann–Whitney U tests and Kolmogorov–Smirnov tests for each action potential feature. These results are added to the Results Section (Lines 187–196) for the distributions shown in Fig. 2D. The additional analyses confirm that the model and data distributions are not significantly different.

—

Comment: L189: please expand the discussion on ghostbursting and doublet firing: it is unclear how each are defined and arise from periodic orbit bifurcations: Fig 4D-3 even says "ghostbursting in the form of doublets".

Reply: We have revised the Introduction (Lines 61–69) to provide a clear definition of ghostbursting, noting that it refers to a burst mechanism first described in ELL pyramidal cells of weakly electric fish by Doiron et al. (Doiron et al., 2002; Doiron et al., 2001). By dividing the model into *slow and fast subsystems*, it was shown that ghostbursting arises when burst termination transitions from period-one to period-two firing in the fast subsystem, causing the burst trajectory to be re-injected near the ghost of a saddle-node bifurcation of fixed points; this gives rise to irregular short bursts (Doiron et al., 2002; Doiron et al., 2001). In the Discussion, we now explain that doublets are a special case of ghostbursting in which bursts contain only two spikes, and that both patterns emerge from the same bifurcation structure (Lines 552–561). We also clarify that while ghostbursting dynamics *in the full system* are governed by saddle-node and period-doubling bifurcations, these structures are not unique to ghostbursting and occur in other classes of bursting neurons (Osinga et al., 2012).

—

Comment: L555: Why is the NMDA driving force written as $(\mathrm{V}-\mathrm{V}_{\mathrm{Ca}})$? Standard NMDA reversal is ~ 0 mV. If this term refers to Ca^{2+} influx through NMDA channels, please clarify that explicitly.

Reply: In our model, the NMDA current does not include the Na^+/K^+ components of the mixed conductance but instead represents only the Ca^{2+} component. As such, its reversal potential is V_{Ca}. In the revised Methods, we now explicitly state that I_{NMDA} denotes only the Ca^{2+} component of the NMDA conductance, since dendritic Ca^{2+} influx is the slow variable that activates SK channels and shapes bursting in our model. Accordingly, we use $(V - V_{Ca})$ as the driving force. The Na^+/K^+ components of the NMDA current (which would yield a reversal potential near 0 mV for the full NMDA conductance) are not accounted for explicitly in the model as they do not contribute significantly to the dynamics analyzed in this study. We added this clarification in the Methods to resolve the potential confusion (Lines 797–802).

—

Comment: Fig. 5A: The dashed blue lines are not explained in the caption – presumably unstable periodic orbits? It is also unclear what happens to the orbits emerging from HB6 as I_{ext} increases; the branch appears to vanish without a visible bifurcation, despite persisting beyond HM2. Similarly, the period-2 orbits from PD1 and PD2 seem to disappear without a clear mechanism. The HR bifurcation diagram would benefit from clearer labelling of key regions and bifurcations, and improved visual design – for instance, use shading instead of dashed boxes for insets, and revise colour choices for better contrast.

Reply: There are two branches (previously labeled envelopes – see response to Minor Comment 12) of limit cycles: (1) A stable one emerging from a supercritical Hopf HB3 and losing stability at a PD1, representing the emergence of multistable periodic orbits; this branch eventually terminates at a homoclinic bifurcation (HM3). (2) An unstable one (represented by blue dashed lines) emerging from a subcritical Hopf HB4 and terminating at subcritical Hopf HB5. The folds within this region represent saddle-node of periodics. We did not focus our analysis on this regime because it is unphysiological, i.e., irrelevant to ghostbursting. The relevant region is the one enclosed by the box encompassing HB1 and PD2. We have now included these points from the comment in the caption of Fig. 5 and addressed them in the Results (see "A simplified phenomenological model of burst firing based on a modified Hindmarsh-Rose model" for changes).

—

Comment: Fig. 5B: It is difficult to follow the f–I curve. Starting from $I = 0$ (for which the system seems monostable, even though it is hard to assess that given the previous comment) and tracking the quiescent branch (zero frequency) for increasing I, from the bifurcation diagram oscillations should emerge just before $I = 0.5$ (at HB1), but the f–I curve remains flat beyond $I = 0$. This conflicts with Fig. 5C, which shows tonic firing at $I = 0.4$. If this discrepancy is due to multistability, it should be explicitly mentioned and clearly supported by the bifurcation diagram. Please revise the figure and caption to ensure consistency and clarify the dynamic regimes being shown.

Reply: We thank the reviewer for bringing up this comment. The model indeed exhibits multistability depending on the initial conditions. In the revised version, we simulated the model with different initial conditions and replotted the f–I curve with a higher sampling rate with respect to I. The revised panel (Fig. 5B) shows the transition to oscillation starting from $I \approx 0.5$.

—

Comment: L247–250: Please clarify what kind of multistability is being referred to. The bottom inset in Fig. 5A suggests that the stable periodic orbit (solid green) emerges from the stable equilibrium at HB3, so it cannot coexist with it – i.e. not multistability between silence and tonic firing. Is the multistability between silence/tonic and the period-2 orbits from PD2 (in adjacent I ranges)? Or with the large-amplitude (light green) orbits emerging from the torus bifurcation? This region is hard to interpret (see earlier comments on Fig. 5A). Please revise this section carefully for clarity. The terminology and schematic representations of multistable regimes in [Marin, B., Barnett, W. H., Doloc-Mihu, A., Calabrese, R. L., & Cymbalyuk, G. S. (2013). High prevalence of multistability of rest states and bursting in a database of a model neuron. PLoS Computational Biology, 9(3), e1002930] could be of use. Consider adding a schematic summary of the dynamical regimes (e.g. quiescent, tonic, bursting) across parameter space to aid interpretation of the bifurcation diagrams.

Reply: As the reviewer correctly noted, the branch of stable periodic orbits (displayed as amplitude bounds) arises from a supercritical Hopf bifurcation; it expands to the left and does not overlap with the stable branch of equilibria. The periodic orbits of this branch subsequently undergo period-doubling bifurcation at PD1, leading to the emergence of multistable periodic orbits. This secondary periodic branch does overlap with the stable equilibrium branch, thereby further broadening the regime of multistability. We have highlighted this behavior in the Results Section (Lines 360–396). As emphasized in our response to Comment 7, it is important to note that this regime is unphysiological.

—

Comment: Fig. 6, L301: "reproduces realistic spiking behavior" is vague. Were parameters in the HR model optimised to fit the data? What would count as a failure or unrealistic firing? Please clarify your criteria.

Reply: We have revised the Results Section (Fig. 6, Lines 435–441) and Methods (Lines 963–973) to define this explicitly. Specifically, parameters of the modified HR model were optimized for each cell using our multi-objective loss function, and we considered fits with loss < 0.1 and non-significant Kolmogorov–Smirnov tests ($p > 0.05$) to reproduce realistic spiking behavior.

—

Comment: L483, elsewhere: This study does not rely on "analytical tractability," nor does it include a systematic evaluation of model efficiency. Please avoid such statements unless they are supported by actual analysis or data.

Reply: Our intention was not to suggest that we had performed a systematic analysis of model efficiency, but rather to emphasize that the modified HR model is more compact than the biophysical model and can still reproduce the main firing patterns. We have revised the text accordingly, namely, instead of referring to "analytical tractability and computational efficiency," we now state that the modified HR model "provides a more compact alternative to the biophysical model" and clarify its potential use for network-level simulations in the future (Lines 705–710, and elsewhere in the manuscript).

—

Comment: The manuscript models background input as additive current noise, but under in vivo-

like synaptic bombardment, noise is typically conductance-based and thus multiplicative in nature (e.g. Destexhe et al., 2003; Richardson, 2004). Please clarify whether the model approximates this, and whether the implications of state-dependent noise were considered.

Reply: We thank the reviewer for raising this point. We acknowledge that a more biophysically detailed representation of synaptic bombardment to model synaptic inputs as fluctuating conductances, which results in state-dependent (e.g., multiplicative) noise, has been previously done (Destexhe et al., 2003; Richardson, 2004). However, in our study, we opted for an additive current-based approximation, which has been widely used in models of *in vivo*–like high-conductance states and shown to reproduce realistic firing statistics across cell types (Brown et al., 1999; Fellous et al., 2003; Fourcaud and Brunel, 2002; Lindner et al., 2003). Importantly, our goal was not to capture detailed synaptic biophysics but to incorporate background fluctuations sufficient to reproduce the variability and bimodality observed in ELL pyramidal cell recordings. We have now added this to the Discussion (Lines 595–603) to clarify this modeling choice and to note explicitly that while conductance-based noise may introduce additional state-dependent effects, the additive approximation used here provides a reasonable and widely accepted simplification for studying spiking variability *in vivo*.

Minor

Abstract

Comment: "calcium handling" sounds vague.

Reply: Fixed.

Summary

Comment: "This simplified phenomenological model successfully captured burst firings" sounds awkward: use either "bursting" or "burst firing" (singular).

Reply: Fixed.

Comment: "validate simplified frameworks for studying population-level dynamics" the link between what has been discussed in the paper and population dynamics is not entirely clear.

Reply: As suggested, it has been removed.

Main text

Comment: L3: "given neural class" please clarify.

Reply: We have now revised the Introduction to clarify that even neurons of the same anatomical or functional type can display diverse spiking activity patterns (Lines 21–24).

Comment: L35: please clarify "dendritic failure".

Reply: We have revised the text to clarify that "dendritic failure" refers specifically to the failure

of dendritic spike backpropagation that normally sustains burst firing (Line 59–60, and elsewhere in the manuscript)

Comment: L49: "constrained its parameter regimes" sounds off - maybe parameter sets?

Reply: Corrected.

Comment: L52: Hindmarsh-Rose is a model, not a formalism - suggest avoiding that term.

Reply: Fixed.

Comment: L53: "significant advantages for modeling population coding. . . " feels overstated. The HR model is certainly efficient as a point neuron with mostly polynomial dynamics, but the actual impact on large-scale simulations is not demonstrated here, especially given the sigmoid dynamics in the fourth variable.

Reply: We originally intended to highlight the computational efficiency of the modified HR model, which makes it a suitable framework for *future* studies of population coding. We have revised the Introduction (Lines 95–97) and the Abstract to be more concise.

Comment: L57, 58: just one of the black arrows corresponds to EA-¿EEL.

Reply: Fixed.

Comment: L63: which kind of stimulation? current clamps?

Reply: We have revised the sentence to clarify that the recordings were performed under baseline conditions, in the absence of external sensory stimulation, with animals housed individually in isolated tanks (Lines 109–113).

Comment: L63: "(Fig. 1A, left; see Methods for details)" shouldn't it be 1A, *right*?

Reply: Fixed.

Comment: L111: "identify the physiologically relevant parameter ranges in vivo" is a bit clunky - suggest rephrasing for clarity.

Reply: We have revised the sentence for clarity. It now reads: "To assess the performance of our model and constrain its parameters to physiologically relevant ranges in vivo, we fitted action potential features from simulations to those extracted from intracellular recordings, selecting parameter values that reproduced experimental features to ensure the model remained realistic (Lines 172–176).

Comment: L168 "how their effect on" ?

Reply: We have revised the sentence for clarity. It now reads: ". . . to understand how they affect spiking activity."

Comment: L170: please define "ghostbursting".

Reply: We have now defined "ghostbursting" in the Introduction (Lines 61–69).

Comment: L182: "two envelopes of stable limit cycle" is clunky and conceptually off - the stable limit cycle branch emerges from the Hopf bifurcation; the upper/lower bounds are just amplitude envelopes shown in the bifurcation plot.

Reply: We have revised the manuscript throughout to avoid the use of "envelopes" and now use the terminology "branches". In the Results and figure captions, we now describe the bifurcation diagrams as showing the branches of periodic orbits, and we clarify that it is the periodic orbit (not the "envelope") that undergoes period doubling. These corrections have been applied across the text, figures, and captions (see "A simplified phenomenological model of burst firing based on a modified Hindmarsh-Rose model").

Comment: L185: similar as above: it is not "the envelopes" that undergo period doubling, but the periodic orbit.

Reply: Corrected, as suggested.

Comment: L201 "boundaries of *the* bursting region": add article

Reply: Fixed.

Comment: L224, 230, 350, 632: Again, I do not think Hindmarsh Rose is a "formalism": it is just a model.

Reply: Fixed.

Comment: L236: "gamma-frequency burst oscillations" have not been defined or mentioned before: are those observed in vivo?

Reply: The phrase "gamma-frequency burst oscillations" referred to ghostbursting dynamics previously described in ELL pyramidal cell models (Doiron et al., 2002; Doiron et al., 2001). To avoid confusion, we have removed this terminology throughout the manuscript and now refer more generally to "burst oscillations" or "irregular bursting dynamics," which are consistent with our data and models.

Comment: L244 "a branch of *an* unstable equilibria": remove "an"

Reply: Fixed.

Comment: L246: see discussion on the use of "envelopes" for L182; please revise elsewhere as well.

Reply: As mentioned in response to previous comments, we rewrote this section entirely to avoid the use of "envelopes".

Comment: L280 "we have added extracellular noise term": missing article

Reply: Fixed.

Comment: L332 "Specifically, it exhibits a rightward shift" do you mean skewness?

Reply: We meant that the ISI distribution of the classic HR model is displaced toward longer

intervals compared to the experimental data, i.e., a rightward shift in location rather than a change in skewness. We have revised the text (Lines 494–495) to clarify this point.

Comment: L358:"dendrites This": missing period.

Reply: Fixed.

Comment: L429: could multistability also lead to dysfunctional regimes? (see eg [Marin, B., Barnett, W. H., Doloc-Mihu, A., Calabrese, R. L., & Cymbalyuk, G. S. (2013). High prevalence of multistability of rest states and bursting in a database of a model neuron. PLoS Computational Biology, 9(3), e1002930]

Reply: We thank the reviewer for this suggestion. Indeed, multistability can in some cases contribute to dysfunctional regimes when neurons switch unpredictably between qualitatively different attractors. For example, Marin et al. (2013) reported that such multistability between rest states and bursting can lead to irregular or unstable firing patterns in model neurons. While our study did not explicitly investigate pathological dynamics, the presence of chaotic multistable regimes in the modified HR model suggests that similar mechanisms could underlie transitions to dysfunctional activity in other systems. We have added a statement in the Discussion (Lines 698–700) to acknowledge this possibility and cite Marin et al. (2013).

Comment: L576: Does Eq. [14] truly correspond to an alpha function? It looks like a single exponential decay, without the rising phase typical of alpha functions.

Reply: We have corrected the equation to correctly describe the equation (Eq. 14; Line 820).

Comment: L611 "[...] the power spectra [...] exhibit a power law spectrum": rephrase

Reply: We have rephrased the sentence. It now reads "synaptic inputs have $1/f^{\beta}$ power spectra, where β determines the exponent of steepness of the slope of the power spectrum" (Line 845).

Comment: Eqs. 29-33 should use SDE notation: $dx = f(x)dt + \sigma dW$. Writing $dx/dt = f(x) + dW$ is not mathematically valid, since dW/dt is undefined. Also, the dW_i are different for each variable. See next comment as well.

Reply: We have revised Eqs. 29—33 to the Itô SDE form, writing them as $dx = f(x), dt + \sigma, dW(t)$ to fix the issue. Each state variable (V, y, z, u) is now associated with its own independent Wiener process (Lines 875–888).

Comment: Line 655: dWi is not a Wiener process, but the differential of one, i.e. stochastic increments. Suggest clarifying that Wi(t) are Wiener processes and dWi are their increments used in the SDEs.

Reply: We have clarified this point in the revised text by explicitly stating that $W_i(t)$ are independent standard Wiener processes, while $dW_i(t)$ are their stochastic increments used in the SDEs. We also added the standard Itô properties ($\mathbb{E}[dW_i] = 0$, $\mathrm{Var}[dW_i] = dt$, $\mathbb{E}[dW_i dW_j] = 0$ for $i \neq j$) to avoid ambiguity (Lines 882–885).

Comment: L725: "Model simulations were carried out using the Euler-Maruyama method (Kloeden et al., 1992)as the integration solver of the stochastic models.": awkward phrasing.

Reply: We have revised the sentence for clarity. It now reads: "Stochastic model simulations were integrated using the Euler–Maruyama method (Kloeden and Platen, 1992)" (Line 1023).

Comment: Fig. 1A: "higher brain area" sounds vague. Consider "higher-order brain region" or a specific anatomical label.

Reply: Corrected.

Comment: Fig. 1B: The phase plane plot seems of limited value - consider replacing it with a zoomed-in view of superimposed spikes, which would better illustrate spike shape variability. Also, the Vm trace appears low-resolution (at least in the PDF), making it hard to assess waveform features like ADPs and AHPs.

Reply: We have now revised the figures to improve clarity.

Comment: L757 "for a representative electrophysiological data from an ELL pyramidal cell.": "a representative electrophysiological data" is ungrammatical. Suggest "representative data" or "a representative recording".

Reply: Replaced with representative data (Figure caption 1B).

Comment: Fig 2C: maybe zoom around spike so that features in B are be more visible? try playing with transparency / line widths so that experimental traces are more discernible?

Reply: We have addressed this by adding a zoomed-in inset to Fig. 3A to show spikes from recordings and model simulations during an example burst.

Comment: L765: Gray box? Markov model? Please revise figure / legend.

Reply: We have removed the description from the figure legend and refer to the Methods Section for a detailed explanation (Figure caption 2A).

Comment: L773: (rough$_v$)$- >$ ($Trough_v$)

Reply: Fixed.

Comment: L777: "modeling simulation" -¿ model simulation / simulation of the model

Reply: Fixed.

Comment: L778: Stray comma/parenthesis after "threshold$_v$"$.Also, \backslash rough_v$"$- > trough_v$

Reply: Fixed.

Comment: Fig 3A: same panel as in fig 1B; all Vm traces look low-res and hard to interpret - the only thing I can almost clearly extract from these plots is the same information provided by the rasters in B (spike times).

Reply: We have decreased the line thickness of the plots to half and added a zoomed-in inset of membrane potential traces to Fig. 3A for data and model, improving clarity.

Comment: Fig 3C, 6C: consider using unfilled circles (or smaller markers?); also, pay attention to label / scale fontsizes throughout the figure

Reply: Corrected.

Comment: Fig 4A, right: aren't PD and PD1 the same bifurcation? also, where is PD2 in the top branch? it is hard to follow periodic orbit branch at this zoom level, maybe it would be helpful to show a zoomed in version of just one part (max or min) of it?

Reply: We thank the reviewer for pointing this out. The labels were missing on the top branch (max) and have now been corrected (Fig. 4A).

Comment: L800: the model undergoes bifurcations (remove "points")

Reply: Fixed.

Comment: L802: "regimes of behavior"?

Reply: This terminology is commonly used to describe regions of parameter space in which distinct dynamical behaviors can be observed.

Comment: L803: There is only one "bounding box"; consider rephrasing as "dashed box" or "highlighted region".

Reply: It is rephrased to the "highlighted region" (see Figure caption 4A).

Comment: L804/807: "the two parameter bifurcation ....?" missing "diagram"?

Reply: Fixed (see Figure caption 4B and C).

Comment: L805/808: "highlighting the various regions of behavior that the model possesses" clunky.

Reply: It has been replaced with "showing the distinct dynamical regimes of the model" to be more precise (See Figure caption 4B and C).

Comment: L806: "Inherently multistable" is vague. Multistability arises from coexisting attractors, not as an inherent property of a region bounded by a bifurcation set. The rest of the sentence ("it overlaps with other regions") is also confusing. Please clarify what regions are meant and how this relates to multistability.

Reply: This has been rewritten to avoid confusion (see Figure caption 4B).

Comment: L811: different values of Iapp, gSK and gNMDA, right? maybe refer to the points 1,2,3,4 highlighted in parameter space?

Reply: Fixed.

Comment: Fig 5: Is any part of the model actually dimensional? You use a.u. for the V variable and for I, but seconds and Hz for time and frequency.

Reply: The modified HR model is formulated in non-dimensional units for the state variables (V, y, z, u) and for the applied current (I_{app}), which is why these are reported in arbitrary units (a.u.) in Fig. 5. Time, however, was explicitly scaled to seconds in order to facilitate comparison with the in vivo recordings, and firing frequency was therefore reported in Hz. Thus, the model is non-dimensional with respect to voltage and current, but dimensional with respect to time. We have clarified this point in the Methods (Lines 884–888).

Comment: Fig 6 and others: would decreasing line widths for all time series lead to clearer plots?

Reply: The linewidths of all figures have been reduced to half their original values.

References

Bastian, J., & Nguyenkim, J. (2001). Dendritic modulation of burst-like firing in sensory neurons. *Journal of Neurophysiology*, *85*(1), 10–22. https://doi.org/10.1152/jn.2001.85.1.10

Brown, D., Feng, J., & Feerick, S. (1999). Variability of firing of hodgkin-huxley and fitzhugh-nagumo neurons with stochastic synaptic input. *Physical Review Letters*, *82*(23), 4731.

Chacron, M. J., & Bastian, J. (2008). Population coding by electrosensory neurons (20080206th ed.). *Journal of Neurophysiology*, *99*(4), 1825–1835. https://doi.org/10.1152/jn.01266.2007

Destexhe, A., Rudolph, M., & Pare, D. (2003). The high-conductance state of neocortical neurons in vivo (2003/09/03). *Nature Reviews Neuroscience*, *4*, 739–751. https://doi.org/10.1038/nrn1198nrn1198pii

Doiron, B., Laing, C., Longtin, A., & Maler, L. (2002). Ghostbursting: A novel neuronal burst mechanism (2002/04/05). *J. Comput. Neurosci.*, *12*, 5–25. https://doi.org/10.1023/a:1014921628797

Doiron, B., Longtin, A., Turner, R. W., & Maler, L. (2001). Model of gamma frequency burst discharge generated by conditional backpropagation (2001/10/16). *Journal of Neurophysiology*, *86*(4), 1523–1545. https://doi.org/10.1152/jn.2001.86.4.1523

Fellous, J.-M., Rudolph, M., Destexhe, A., & Sejnowski, T. J. (2003). Synaptic background noise controls the input/output characteristics of single cells in an in vitro model of in vivo activity. *Neuroscience*, *122*(3), 811–829. https://doi.org/10.1016/j.neuroscience.2003.08.027

Fourcaud, N., & Brunel, N. (2002). Dynamics of the firing probability of noisy integrate-and-fire neurons. *Neural computation*, *14*(9), 2057–2110.

Kloeden, P. E., & Platen, E. (1992). Stochastic differential equations. In *Numerical solution of stochastic differential equations* (pp. 103–160). Springer. https://doi.org/10.1007/978-3-662-12616-5_4

Lindner, B., Longtin, A., & Bulsara, A. (2003). Analytic expressions for rate and cv of a type i neuron driven by white gaussian noise. *Neural computation*, *15*(8), 1761–1788.

Loewenstein, Y., Mahon, S., Chadderton, P., Kitamura, K., Sompolinsky, H., Yarom, Y., & Häusser, M. (2005). Bistability of cerebellar purkinje cells modulated by sensory stimulation. *Nature neuroscience*, *8*(2), 202–211.

Osinga, H. M., Sherman, A., & Tsaneva-Atanasova, K. (2012). Cross-currents between biology and mathematics: The codimension of pseudo-plateau bursting. *Discrete and continuous dynamical systems. Series A*, *32*(8), 2853.

Richardson, M. J. (2004). Effects of synaptic conductance on the voltage distribution and firing rate of spiking neurons. *Physical Review E—Statistical, Nonlinear, and Soft Matter Physics*, *69*(5), 051918.
