# Supplementary material for: In vivo neural activity of electrosensory pyramidal cells: Biophysical characterization and phenomenological modeling
Source: PLoS Comput Biol. 2025 Nov 17;21(11):e1013711. doi: 10.1371/journal.pcbi.1013711 (PMC12633970; doi:10.1371/journal.pcbi.1013711)
Supplement: Attachment — Submitted filename: Reviewer comments.pdf [file pcbi.1013711.s009.pdf]

The current manuscript investigates how burst firing in electrosensory pyramidal cells of the electrosensory lateral line lobe (ELL) in the weakly electric fish *Apteronotus leptorhynchus* is generated and modulated. The authors combine intracellular recordings with computational modeling to study the activity of these neurons.

They first constructed a detailed biophysical, two-compartment model based on in vivo data, incorporating intrinsic ionic conductances, SK channel activation, calcium dynamics, and stochastic synaptic inputs. Using bifurcation analysis, they show that these parameters govern transitions between quiescent, tonic, and bursting firing modes. The authors further developed a phenomenological model based on the Hindmarsh-Rose (HR) formalism, demonstrating that this modified HR model can reproduce ELL neuronal activity. Both models are compared and validated against the in vivo recordings. Finally, a parameter sensitivity analysis highlights the importance of slow adaptation and stochastic input in shaping ELL neurons' spiking patterns and provides a compelling hypothesis to explain the differences between in vivo and in vitro recordings of these neurons.

The model is both detailed and valid, and compared rigorously with the in-vivo data that have been recorded for the project. However, there are some limitations on the experimental side that might greatly weaken some of the conclusions made in the manuscript.

Major points

- The neurons measured in the in-vivo model animals are described as being recorded in absence of stimulation. However, the authors specify that "*ELL pyramidal cells receive direct ascending inputs from electroreceptor afferents*": were there any controls in the experimental setup that made sure electroreceptors were not activated, or that there was no electric field that could have been detected? If not, could the authors please explain why it is not necessary to make such control? While my expertise is with rodens models, I understand that weakly electric fish self-generate electric fields and use them to sense nearby objects: their electroreceptors being activated by these self-generated currents. Were these currents being measured, or can we be sure they could not have been present due to the condition the animals were in?

  If potential external sensory stimuli can not be ruled out, this could mean that the importance of intrinsic noise could have been (potentially greatly) overstated, therefore impacting the main conclusions of the study.

- The experimental part of the study suffers from a low number of animals the recordings were taken from. While a preferable solution would be to improve the number of recorded fish, I would at least recommend the authors to clearly show the variability of the data across the 3 fish they recorded from.
  One way to do this would be to plot AP-related electrophysiological characteristics for the 3 fish. Showing that these characteristics are consistent across animals would greatly improve the reliability of the in-vivo results.

Further, and closely related to the previous point, a common source of biased results when averaging over all the cells from multiple animals together, happens when the number of recorded cells greatly fluctuates between animals: can the authors please specify how many cells they recorded from each animal?

Minor points

- Since one of the objectives of the model is to capture bursting behavior, I believe it would be beneficial to show a comparison between a typical burst as recorded in-vivo, and a burst generated by the model. Though this is partly done in Figure S3, I believe this is important to show in one of the main figures. The most straightforward way to do this would probably be to make a small addition to Figure 3, showing via a “zoom in”, the typical shape of a recorded vs generated burst.
- In Figures 3 and S3, the authors plot the electrophysiological characteristics of recorded vs generated action potentials, and associated action potential trains. While it is clear that the model correctly represents the experimentally recorded data, it would be beneficial to also compare the variability between the two samples. A way to do this might be to add error bars (on both axis) to these plots, or make separate plots in addition to Figure S3.
- In Figure S1B, the authors plot the excitatory neurons mean firing rate, however, from the context it is difficult to understand if the firing rate is relative to only bursts, or to the entire recordings. Further, the length of the recordings is not specified (or I can not find it in the methods or figure legends)
- Flow and readability: at line 36, the authors write: “*The in vivo recordings, on the other hand, show markedly different firing patterns, typically consisting of only 2–3 spikes per burst on average, with no evidence of a somatic DAP or burst AHP*”.
  The in vitro vs in vivo comparison is a key point of the paper, and the authors do mention DAP and AHP, however, adding a clearer description of how many APs are typical in an in-vitro burst would help the reader in following the text.
